# Experimental Studies on the Centrifugal MQL-CCA Method of Applying Coolant during the Internal Cylindrical Grinding Process

**DOI:** 10.3390/ma13102383

**Published:** 2020-05-22

**Authors:** Krzysztof Nadolny, Seweryn Kieraś

**Affiliations:** 1 Department of Production Engineering, Faculty of Mechanical Engineering, Koszalin University of Technology, Racławicka 15-17, 75-620 Koszalin, Poland; 2 Wartsila Poland Sp. z o.o., Łużycka 2, 81-537 Gdynia, Poland; seweryn.kieras@wartsila.com

**Keywords:** internal cylindrical grinding, minimum quantity lubrication, compressed cooled air, 100Cr6 steel, tool life

## Abstract

This paper presents the results of experimental research concerning the possibility of supporting the cooling function during internal cylindrical grinding using the minimum quantity lubrication (MQL) method by additional delivery of a compressed cooled air (CCL) stream. The article presents a description of a hybrid method of cooling and lubrication of the grinding zone integrating centrifugal (through a grinding wheel) lubrication with the minimum quantity of lubricant and cooling with a compressed cooled air stream generated by a cold air gun (CAG). The methodology and results of experimental studies are also presented in detail, with the aim of determining the influence of the application of the hybrid method of cooling and lubrication of the machining zone on the course and results of the internal cylindrical grinding process of 100Cr6 steel in comparison with other methods of cooling and lubrication, as well as compared with dry grinding. The research results obtained using the described hybrid method of cooling and lubrication of the grinding zone are related to the results obtained under the conditions of centrifugal MQL method, cooling with a stream of CCA, cooling and lubrication with a stream of oil-in-water emulsion delivered using the flood method, and dry grinding. The efficiency of the grinding process is evaluated (based on the average grinding power *P_av_*, grinding wheel volumetric wear *V_s_*, material removal *V_w_*, and grinding ratio *G*), along with the thermal conditions of the process (based on the analysis of thermograms recorded by infrared thermal imaging method), the textures of machined surfaces (based on microtopography measured by contact profilometry), the state of residual stress in the surface layers of workpieces (determined by X-ray diffraction method), and the state of the grinding wheels’ active surfaces after grinding (based on microtopography measured by laser triangulation and images recorded with a digital measuring microscope). The obtained results of the analyses show that the application of the hybrid method allows for the longest wheel life among the five compared grinding methods, which is about 2.7 times the life of grinding wheels working under the flood cooling and centrifugal MQL methods, and as much as 8 times the life of grinding wheels working under the conditions of CCA only and dry grinding.

## 1. Introduction

The processes of abrasive machining with bonded abrasive tools can be discussed as a tribosystem. The interdependencies between the elements of this system (workpiece, abrasive grains, bonds, and machining environment) determine the tribological processes in the contact area. The combined effects of the processes strongly influence the course and results of abrasive machining. These processes can be divided into five basic groups: contact processes [1], friction processes [2,3], abrasive tool wear processes [2,3,4,5,6], workpiece wear processes [3], and lubrication processes [3,7]. Among the abovementioned processes, lubrication processes of the tool–workpiece contact zone have a key influence on the conditions of abrasive machining, as they affect the physicochemical processes of the contact. In general, dry machining and machining with the use of a coolant, lubricant, and antiadhesive agent can be distinguished.

The literature describes a large number of methods of delivering coolants in the form of liquids, gases, solid lubricants, and antiadhesive mediums to the grinding zone. In recent years, growing environmental awareness has led to increasing attention being paid to developing methods to reduce coolant usage in machining processes, including grinding. Techniques are being developed to minimize the coolant expenditure, which can be described as hybrid methods, as they simultaneously deliver several types of coolant, lubricant, and antiadhesive agents. Most often, the transport medium is compressed air. The best-known hybrid methods of cooling and lubrication of the grinding zone are:

- Minimum quantity lubrication (MQL) [8,9,10,11,12,13,14,15,16,17,18,19,20,21,22,23,24,25,26,27,28,29];
- Minimum quantity cooling (MQC) [1,30];
- Minimum quantity cooling lubrication (MQCL) [31,32,33,34,35,36,37,38];
- Cold air MQL (CAMQL) [24,39,40];
- Cold air and oil mist (CAOM) [41,42,43,44,45];
- Using solid lubricants and antiadhesives [28,46,47].

Due to its ease of use and versatility, as well as its good functionality, the CAOM method, which integrates the simultaneous use of MQL and cold air guns (CAG), appears to be particularly beneficial. The two methods are presented in more detail below.

The MQL method was developed in order to ensure the most favorable conditions for the implementation of machining, while minimizing expenditure on coolants. In the MQL systems, oil is sprayed under the influence of compressed air energy onto the grinding wheel active surface (GWAS) and the workpiece surface. In the literature sources [22,23,48], a description is given of the MQL as a very important alternative to dry grinding, describing it as a near dry grinding (NDG) or minimum coolant grinding (MCG) technique. Even a small amount of liquid coolant entering the contact zone between the GWAS and workpiece surface can have a positive effect on the efficiency of the grinding process. Such an approach was the basis for the development of MQL systems aimed at reducing the environmental risk and disposal costs by limiting the amount of liquid coolant used [8,14,18,27,49,50,51].

In the MQL method, the liquid coolant output of 7.2–97.2 mL/h (nearly 1000 times less than in the flood method) is used, while at the same time the liquid coolant is delivered precisely to the GWAS contact zone with the machined surface [21,26,52]. The air–oil aerosol covers the GWAS and the workpiece surface, cooling them down and applying a lubricating oil film layer on the machined surface. This reduces the friction force between the abrasive grains and the workpiece surface, and also reduces the grinding force *F* and the amount of heat generated [53,54,55].

In the MQL method, the lubrication function is usually provided by oil, whereas the cooling function is provided mainly by compressed air. This very small amount of liquid coolant delivered to the grinding zone significantly reduces the friction in the GWAS contact zone with the machined surface and limits the adhesion of the grinding products to the grinding wheel [56,57].

The amount of heat received by air is very limited and its ability to conduct heat is insufficient to efficiently cool the grinding zone [29]. The research performed by Silva et al. [22] shows that the MQL method provides lubrication at a higher level than the flood method, however the cooling function of the GWAS contact zone with the workpiece surface is much less effective. Table 1 presents the values of thermal capacity of the media used in the MQL method (air and oil) in comparison with the thermal capacity of water.

In the research described by Sadeghi et al. [19], machined surfaces of steel in a hardened state after grinding with the MQL method were characterized by the lowest *Ra* roughness parameter value among the considered cooling methods. Additionally, Tawakoli et al. [48] showed that the use of the MQL method reduces the *Ra* parameter value of the ground surface roughness in relation to flood method and dry grinding.

The application of the MQL method may contribute to the reduction of wear phenomena associated with active cutting vertices on the GWAS, causing them to maintain their sharpness over a longer period of time with respect to dry grinding, and sometimes also with respect to flood cooling (when grinding steel in the hardened state). This may have the effect of reducing the cross-sectional area of chips obtained in the process of grinding with this method and improving the morphology of the workpiece surface [19,22,23,54].

Tawakoli et al. [24] determined the influence of air–oil aerosol parameters obtained with the MQL method on the process of 100Cr6 steel grinding. The study showed that the angle of the air–oil aerosol delivery nozzle outlet has a significant effect on grinding conditions and results.

The application of the MQL method avoids the clogging of intergranular spaces on the GWAS, while the lubrication is carried out on the whole circumference of the wheel, which ensures better slipping at the contact areas of active grains with the machined surface [22]. However, the studies described by Hadad and Sharbati [16] and Li et al. [58] show that similarly to dry grinding, using the MQL method involves a high risk of grinding burns resulting on the workpiece.

The application of cooled compressed air is a constantly evolving field of scientific research and technical applications, and the nozzles used in this method are referred to as cold air guns (CAGs). The method is used in machining processes and in grinding [59,60].

The minimization of the coolant and lubricant output, which takes part in the grinding process, may cause an increase in the workpiece temperature and the formation of thermal defects on the machined surface. Supporting the grinding processes with the application of a stream of compressed cooled air may significantly reduce the temperature in the grinding zone and reduce or completely eliminate the occurrence of thermal defects [41,42].

The equipment used to obtain and supply compressed cooled air is characterized by uncomplicated construction, low purchase cost, and easy operation. The CAG nozzle is a device that creates a stream of compressed cooled air using vortex tubes. In CAG nozzles, the compressed air stream is rotated around its own axis in the vortex tube. This allows one to obtain streams of cold and hot air. The cooled compressed air obtained by means of CAG nozzles can reach temperatures 55 °C lower than those of the air supplying the device [40,60].

The results of the tests described by Ramesh et al. [7] showed a decrease in the value of the grinding force during the grinding process under the conditions of supplying a stream of CCA (with a pressure of 0.3 MPa, air temperature at the nozzle outlet in the range from −30 °C to −35 °C, flow rate of 0.4 m^3^/min) in comparison with the grinding using the flood method, with specific material removal rates of *Q’_w_* = 1.6 mm^3^/s·mm for S45C steel and *Q’_w_* = mm^3^/s·mm for SS304 steel.

Choi, Lee, and Jeong [61] have shown that the application of 4% oil-in-water emulsion for cooling and lubrication of the grinding process allows one to obtain lower values for roughness parameters *Ra* and *Rz* than in the case of delivery of CCA to the grinding zone. The higher value of roughness parameters of the ground surface *Ra* and *Rz* in the case of cooling of the grinding zone with the use of a CAG nozzle may have been caused by the lack of lubrication and the limited possibility of cleaning the workpiece and grinding wheel surfaces, as compared to the process carried out with the use of oil-in-water emulsions.

Choi, Lee and Jeong [41] carried out studies on the effect of CCA delivery on the process of internal cylindrical grinding with a cubic boron nitride (cBN) grinding wheel and Al_2_O_3_ abrasive grains. It was shown that with a decrease in the temperature value of the compressed cooled air stream and an increase in its discharge velocity, the *Ra* and *Rz* roughness parameter values of the machined surface decreased and the occurrence of thermal defects of the workpiece was reduced. Moreover, it was observed that the value of tensile stress occurring on the surface layer of the workpiece after grinding decreased with an increase in the CCA flow rate from the nozzle [41].

Lee and Lee [59] optimized the microgrinding process with the use of compressed cooled air. The optimization of the process made it possible to reduce the specific grinding force *F’* and to reduce the value of the *Ra* roughness parameter of the ground surface, while at the same time maximizing the specific material removal rate *Q’_w_*, thus indicating the great potential of this method.

Cooling the grinding zone with the use of CAG nozzles in a very effective way minimizes the occurrence of grinding defects on the treated surface, and moreover minimizes their application and reduces the negative impact on the PCS environment, which is consistent with the current trend of development of manufacturing techniques [61].

Both of these described methods are combined in the CAOM method, in which the lubrication function realized by the MQL method is additionally supported by the CCA stream generated by the CAG nozzle. As a result, it is possible to avoid the occurrence of unfavorable changes in the structure of the workpiece surface layer in the form of burns [41,42]. The described features of the MQL method and CAG nozzles were the basis for undertaking research, aiming to implement this CAOM method in the internal cylindrical grinding process. To date, such cooling and lubrication methods had been developed only for surface grinding, cylindrical grinding, and shape grinding, processes. As a result, a new hybrid method was developed, in which a patented method of centrifugal air–oil aerosol delivery through the grinding wheel was combined with a CAG nozzle generating CCA [62]. The aim of the presented research was to determine the influence of the application of the hybrid cooling and lubrication method in the grinding zone on the course and results of internal cylindrical grinding of 100Cr6 steel in comparison with other methods of cooling and lubrication, as well as dry grinding. In particular, the study sought to determine the effects of the use of the centrifugal MQL-CCA method on the life of a grinding wheel, the grinding power *P*, volumetric wear of the grinding wheel *V_s_*, material removal *V_w_*, grinding ratio *G*, thermal conditions of the grinding process, roughness and residual stress of the machined surface, and clogging of the GWAS in comparison to the four other varieties of cooling and lubrication conditions. This paper presents a description of a hybrid method of cooling and lubrication of the grinding zone, integrating centrifugal (through a grinding wheel) lubrication with a minimum quantity of lubricant and cooling with a compressed cooled air stream generated by a cold air gun (CAG) (Section 2). Then, the methodology (Section 3.1) and results (Section 3.2) of experimental studies are presented in detail, with the aim of determining the influence of the application of the hybrid method of cooling and lubrication of the machining zone on the course and results of the internal cylindrical grinding process of 100Cr6 steel in comparison with other methods of cooling and lubrication, as well as dry grinding. In the last part of the manuscript (Section 4), detailed conclusions are given in relation to a number of criteria to assess the grinding process.

## 2. Setup of Centrifugal MQL-CCA Method of Coolant Delivery during Internal Cylindrical Grinding Process

While developing the described hybrid method of cooling and lubrication of the grinding zone, the need to support the cooling function (implemented in the MQL method to a very limited extent) was taken into account, along with the problem of collecting chips on the workpiece. The minimum flow of the oil delivery in the form of an air–oil aerosol in the MQL method means that chips and other grinding products are not washed out of the machining zone, which can cause them to end up in the GWAS–workpiece contact zone again, interfering with the machining process. Accumulation of chips close to the grinding zone also increases the risk of clogging intergranular spaces of the GWAS. Accordingly, the described method uses a dual-outlet CCA supply line from the CAG nozzle to the machining zone. One outlet of the line was directed ahead of the grinding zone to cool it, while the other outlet was located directly behind the grinding wheel–workpiece contact zone to blow out the machining products (mainly chips).

The following components of the centrifugal air–oil aerosol delivery system were used to configure the test stand:

- A ZR-K 360° six-nozzle, omnidirectional, minimum quantity lubrication head;
- MQL head supply system, with compressed air and oil from the workpiece spindle side;
- A specially designed grinding wheel arbor;
- The system used to fix the ZR-K 360° head inside the rotating grinding wheel arbor;
- A special ceramic grinding wheel with dimensions of 40 mm × 20 mm × 26 mm, adapted to work with the hollow grinding arbor;
- As a lubricant, an oil was used called Cimtech^®^ MQL from CIMCOOL^®^ Fluid Technology, part of Milacron LLC.

In addition, a Vortec 610 CAG nozzle equipped with a dual-outlet supply line was placed in near the grinding zone, allowing for precise directing of the CCA jet to the desired areas of the grinding zone [63]. The outlets of the CAG nozzle supply line had openings with a diameter of 6.3 mm and the nozzle itself was supplied with compressed air at a pressure of 0.6 MPa. As a result, the CAG reduced the air temperature at the outlet from the supply line to about −5 °C. The Vortec 610 nozzle configured in this way gave CCA with a total output (calculated for two outlets) *Q_CCA_* = 49.8 dm^3^/min (0.00083 m^3^/s).

**Figure 1:**
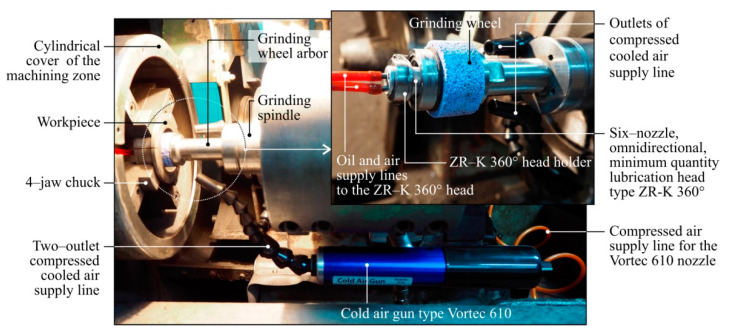
Overall view of the grinding zone with components of the centrifugal MQL-CCA system.

Figure 1 shows the view of the machining zone of the RUP 28P universal grinding machine (Mechanical Works Tarnów SA, Tarnów, Poland) [64], with all components necessary for the grinding process according to the assumptions of the described hybrid cooling and lubrication method.

The research was divided into two parts. In the first stage, simulation tests were carried out, using the computational fluid dynamics (CFD) supported by the finite element method. The aim was to determine the most favorable geometrical and kinematic parameters of the process in terms of the flow of cooling and lubricating media (air–oil aerosol and CCA), as well as heat exchange.

The conditions and results of these simulations were described in detail in the paper [65]. The most advantageous of the variants analyzed in the simulation studies was then selected for experimental research, in which the service life of the grinding wheels was determined with reference to four methods of cooling and lubricating the machining zone.

## 3. Experimental Research on the Internal Cylindrical Grinding Process

The following sections describe in detail the conditions for conducting the experimental tests (Section 3.1) and present the analysis of the obtained results (Section 3.2).

### 3.1. Methodology of Experimental Research

Experimental tests were conducted in the process of reciprocal internal cylindrical grinding using grinding wheels of technical designation 1-40 × 20 × 26 SG/F46L7VTO, made by the team of Prof. Daniela Herman with the use of a ceramic glass–crystalline bond [66,67,68]. Internal surfaces of bearing rings made of 100Cr6 steel (50 ± 2 HRC) were ground. The grinding process was undertaken on a test stand equipped with a RUP 28P universal grinding machine [64].

Five varieties of cooling and lubrication conditions were adopted for the study (four of which were the reference for the newly developed hybrid method):

- Hybrid method of cooling and lubrication of the grinding zone, integrating centrifugal minimum quantity lubrication method and cooling with compressed cooled air stream (abbreviation: *centrifugal MQL + CCA*);
- Centrifugal minimum quantity lubrication (abbreviation: *centrifugal MQL*);
- Compressed cooled air flow cooling (abbreviation: *CCA*);
- Cooling and lubrication of the oil-in-water emulsion applied by the flood method (abbreviation: *flood method*);
- Grinding without cooling and lubrication agents (abbreviation: *dry grinding*).

The components and operating parameters of the air–oil aerosol (MQL) delivery system with compressed cooled air and a CAG nozzle were given earlier in Section 2, describing the centrifugal MQL-CCA method.

The end of the grinding wheel service life in given conditions of cooling and lubrication of the grinding process was determined on the basis of observation of grinding power values, assessment of the degree of GWAS clogging, and also on the basis of detection of thermal defects on the ground surfaces (grinding burns). During the tests, a grinding power gain *ΔP* (which is the difference between the grinding power *P* and spindle electric power at idling speed), material removal *V_w_*, and grinding wheel volumetric wear were recorded. At the end of the tests, the grinding ratio *G* was calculated for the machined surface texture parameters using a Hommel-Tester T8000 contact profilometer (Hommelwerke Gmbh, Villingen-Schwenningen, Germany) and TalyMap Silver 4.1.2 software (Taylor Hobson, Leicester, UK). The assessment of the GWAS conditions after machining was carried out using microtopographs recorded by a Talysurf CLI 2000 measuring system (Taylor Hobson, Leicester, UK) [69]. Furthermore, GWAS images were registered with a Dino-Lite Edge AM7915MZT digital measuring microscope (AnMo Electronics Corporation, New Taipei City, Taiwan) using DinoCapture 2.0 software (AnMo Electronics Corporation, New Taipei City, Taiwan) [70]. The influences of different cooling and lubrication conditions during the grinding process on the grinding wheel and workpiece temperature were also determined by recording thermograms of the grinding zone using a Testo 890 thermal imaging camera (Testo GmbH, Wien, Austria) and IRSoft 3.2 software (Testo GmbH, Wien, Austria). The thermograms were recorded from a fixed position at a distance of approximately 50 cm from the grinding zone. Temperature values derived from them cannot be treated as the real temperatures in the contact zone of the active vertices of abrasive grains with workpiece surface. However, the thermal imaging technique provides reference information and has been used for comparison purposes to assess the differences in thermal conditions under various cooling and lubrication conditions in the machining zone. The uncertainty of the measuring instruments used was not taken into account in the described studies.

**Figure 2:**
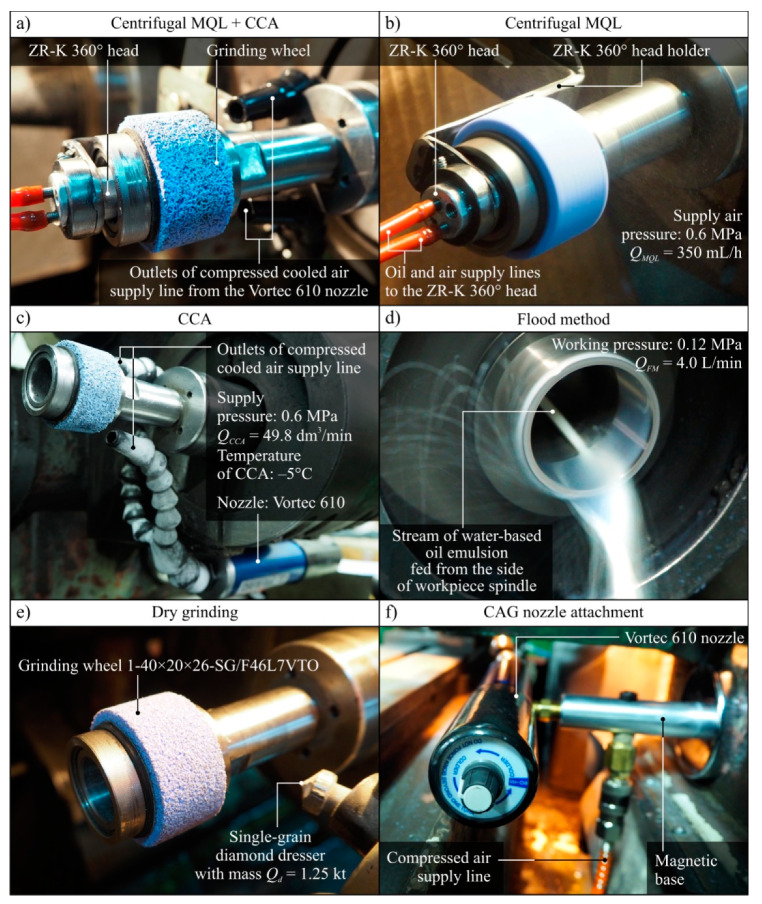
View of the machining zone for the five compared cooling and lubrication conditions: (**a**) centrifugal MQL and CCA; (**b**) centrifugal MQL; (**c**) only CCA; (**d**) flood method; (**e**) without coolants and lubricants (dry grinding); (**f**) fixing the CAG nozzle in the machining zone.

Table 2 gives a synthetic summary of the grinding conditions applied in the described experimental studies. Figure 2 shows views of the machining zone with the components necessary for grinding, using the five compared cooling and lubrication methods.

### 3.2. Results of Experimental Studies and Their Analysis

The results of the experimental studies are described in five steps, regarding the:

- Grinding process efficiency based on the average grinding power *P_av_*, volumetric wear of the grinding wheel *V_s_*, material removal *V_w_*, and grinding ratio *G*;
- Thermal conditions of the grinding process on the basis of infrared thermal imaging measurements;
- Machined surface texture analysis;
- Residual stress in the surface layer of the ground surface determined by X-ray diffraction;
- Condition of the GWAS after grinding based on microtopography and microscopic images.

#### 3.2.1. Evaluation of the Efficiency of the Grinding Process

**Figure 3:**
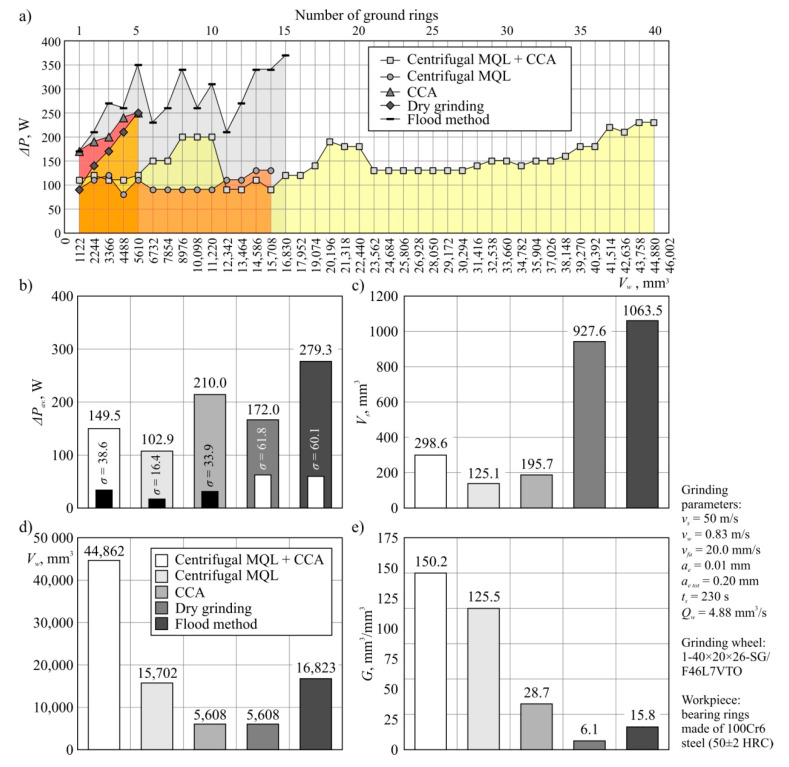
Grinding power gain *ΔP* values and values of parameters describing the tested grinding wheels’ lifespans: (**a**) grinding power gain *ΔP*; (**b**) average grinding power gain *ΔP_av_* with the standard deviation *σ*; (**c**) material removal *V_w_*; (**d**) volumetric grinding wheel wear *V_s_*; (**e**) grinding ratio *G* = *V_w_*/*V_s_*.

The diagrams in Figure 3 show the changes in the value of grinding power gain *ΔP* and parameters describing the service life of the tested grinding wheels (*V_s_*, *V_w_*, *G*) operating under the five cooling conditions. The analysis of the grinding power gain *ΔP* values presented in Figure 3a shows that the application of the hybrid method allowed for the longest grinding wheel life. In such conditions, it was possible to carry out effective machining up to ring number 40 (*V_w_* = 44,862 mm^3^). After grinding of this workpiece, an increase in the clogging of the GWAS was found. When using only centrifugal air–oil aerosol delivery and cooling and lubrication with the traditional flood method, grinding wheel life periods of 15 (flood method) and 14 (centrifugal MQL) rings were obtained, which corresponded to the removal of 16,823 mm^3^ and 15,702 mm^3^ of machined material, respectively (Figure 3d). The end of the life of the grinding wheel working with the flood method was due to the occurrence of grinding burns on the machined surface. During grinding under centrifugal MQL conditions, there was a significant increase in the clogging of the GWAS, which caused the end of the service life. The shortest life span was recorded during grinding with only CCA and in dry grinding. Wheels in such conditions worked effectively only up to ring number 5 (*V_w_* = 5608 mm^3^), after which grinding burns were observed on the machined surface.

By referring the value of the workpiece removal *V_w_* to the total volumetric wear of respective grinding wheels over the entire service life, the grinding ratio *G* was determined, the values of which are shown in Figure 3e. The analysis of the set of parameters describing the durability period of the tested grinding wheels presented in Figure 3 shows that for the assumed parameters of internal cylindrical grinding, the most favorable results were obtained by using the proposed hybrid method of cooling and lubrication of the machining zone. The effectiveness of this method of influencing tribological conditions in the GWAS–workpiece contact zone is mainly explained by the precision of the lubricant supply (centrifugal supply of air–oil aerosol) and the cooling medium (CCA stream directed before and after the grinding zone). Even the use of an abundant stream of oil-in-water emulsion delivered by the traditional flood method with a flow rate of 4.0 L/min did not allow for equally favorable grinding results. As a result, the hybrid method provided about 2.7 times longer service life in relation to the flood and centrifugal MQL methods, and as much as 8 times longer compared to the grinding wheels’ operating time under the conditions of CCA only and dry grinding.

The determined average grinding power gain *ΔP_av_* (Figure 3b) ranged from 102.9 W for centrifugal MQL to 279.3 W for the flood method. The application of the hybrid method resulted in an average *ΔP* value of 149.5 W over a much longer grinding wheel life. This very favorable result can be explained by the limited friction of the dulled cutting vertices of the grinding wheel resulting from the effective penetration of oil into the grinding wheel–workpiece contact zone (fed centrifugally through the pores of the wheel). On the other hand, the CCA stabilized the thermal conditions in the machining zone, reducing the abrasive wear of the active abrasive vertices, which retained their cutting ability over a longer period of time. Both factors led to a reduction in the power expenditure associated with friction. However, the analysis of the diagram in Figure 3a shows a gradual increase in grinding power resulting from the progressive wear of the wheel.

#### 3.2.2. Evaluation of the Thermal Conditions of the Grinding Process on the Basis of Infrared Thermal Imaging Measurements

In order to consider thermovision measurements in the differences resulting from different grinding wheels and workpiece materials, different emission coefficient *ε* values were adopted:

- *ε* = 0.94 for the grinding wheel—the value appropriate for Al_2_O_3_ and glass [71,72];
- *ε* = 0.80 for the workpiece—the value corresponding to the steel surface at 93 °C was taken (the average value from range 0.75 to 0.85 was adopted) [71,72].

For each variety of cooling and lubrication conditions of the grinding zone, several dozen thermograms were recorded during the processing of one workpiece (at intervals of about 10 s), with a total of 2230 thermograms recorded in these tests. For each working cut, the measurement with the highest recorded temperature value was then searched and subjected to further detailed analysis. Additionally, thermograms of the processing zone recorded directly after grinding were analyzed. Such measurement was carried out with the coolant supply switched off and was free of any disturbances resulting from their influence.

**Figure 4:**
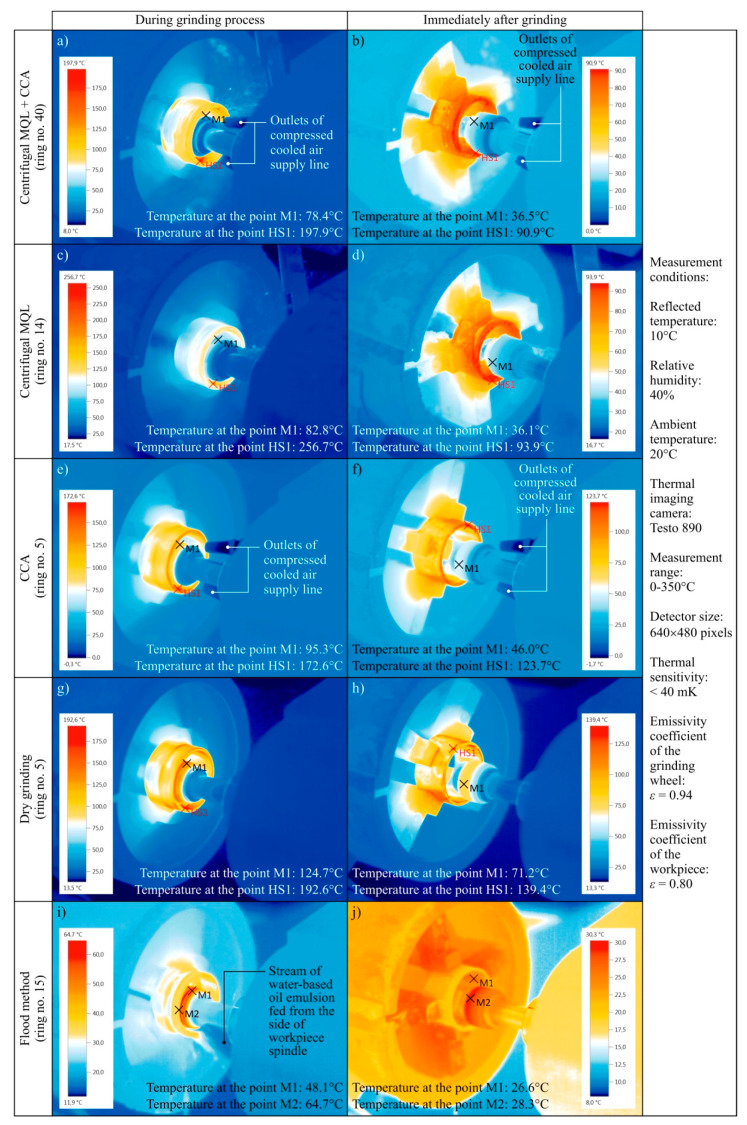
Thermograms recorded during (**a**,**c**,**e**,**g**,**i**) and immediately after grinding (**b**,**d**,**f**,**h**,**j**) of the last workpiece from the grinding wheel service life: (**a**,**b**) hybrid method of cooling and lubrication (centrifugal MQL + CCA); (**c**,**d**) centrifugal MQL; (**e**,**f**) CCA; (**g**,**h**) dry grinding; (**i**,**j**) flood method.

**Figure 5:**
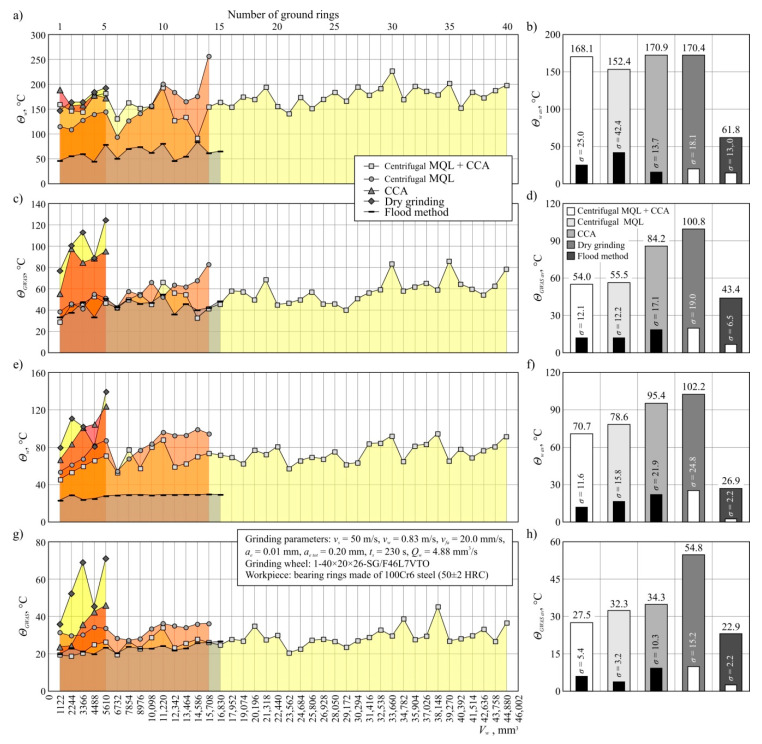
Workpiece and grinding wheel temperature measured during the grinding process (**a**–**d**) and immediately after the grinding process (**e**–**h**): (**a**,**e**) workpiece temperature *Θ_w_*; (**b**,**f**) average workpiece temperature *Θ_w av_* with the standard deviation *σ*; (**c**,**g**) grinding wheel temperature *Θ_GWAS_*; (**d**,**h**) average grinding wheel temperature *Θ_GWAS av_* with the standard deviation *σ*.

Figure 4 shows sample thermograms recorded during and directly after grinding with the use of the five tested varieties of grinding process. This figure shows the results of measurements carried out during the machining of the last ring during the grinding wheel life. Additionally, Figure 5 presents changes in the temperature values of the workpiece *Θ_w_* and the grinding wheel *Θ_GWAS_* during the entire lives of the grinding wheels operating in the five variants, analyzed together with the determined average values of these parameters; Figure 5a–d show the results of measurements carried out during grinding, while Figure 5e–h show the results of measurements carried out after grinding.

The analysis of temperature values determined during grinding in five different cooling and lubrication conditions of the grinding zone (Figure 5a–d) shows that the smallest temperature values of both the workpiece (Figure 5a) and GWAS (Figure 5c) are obtained using the flood method (*Θ_w av_* = 61.8 °C, grinding wheel *Θ_GWAS av_* = 43.4 °C). All other variants from the NDG groups (hybrid method, centrifugal MQL, and CCA itself), as well as dry grinding, were characterized by significantly higher average workpiece temperatures, which ranged from 152.4 °C to 170.9 °C (Figure 5b). The relatively high workpiece temperature values recorded during grinding for all techniques except the flood method may have resulted from a large shaft of sparks (oxidizing chips) emitted from the machining zone and interfering with the measurement results. In the case of the flood method, the sparks were much smaller due to the much higher expenditure of the coolant (oil-in-water emulsion). Referring to the mean temperature of GWAS (Figure 5d), it can be observed that the lowest values of this parameter (except for the flood method) were obtained using the hybrid method (*Θ_GWAS av_* = 54.0 °C) and centrifugal MQL (*Θ_GWAS av_* = 55.5 °C). For grinding with delivery of CCA *Θ_GWAS av_* = 84.2 °C, while in the dry process temperatures as high as 100.8 °C were recorded. It follows that the application of the hybrid method reduces the grinding wheel temperature by half in relation to dry grinding. It is worth noting that a comparable result was recorded with the application of a centrifugal MQL without a CCA delivery.

The beneficial effect of the additional use of a CAG nozzle in CCA delivery was revealed by the analysis of temperature values determined immediately after the grinding process (Figure 5e–h). In this case, the most advantageous (the smallest) values were determined for flood cooling (*Θ_w av_* = 26.9 °C, *Θ_GWAS av_* = 22.9 °C), which did not differ significantly from the temperature prevailing in the abrasive treatment laboratory (about 20 °C). However, comparing the results obtained for a group of methods that are much more advantageous in terms of environmental impact, the newly developed hybrid method may be considered the most advantageous one. The additional CCA flux supporting the MQL method resulted in obtaining about 30% lower value of *Θ_w av_* = 70.7 °C and about 50% lower value of *Θ_GWAS av_* = 27.5 °C in relation to results obtained under dry grinding conditions (*Θ_w av_* = 102.2 °C, *Θ_GWAS av_* = 54.8 °C) (Figure 5f,h).

It should be stressed that these favorable results were obtained by comparing the average values of the described parameters determined for significantly different grinding wheel life periods. Thus, the obtained results of thermographic measurements confirm that it is possible to obtain stable thermal conditions in the grinding zone over long periods of grinding wheel operation using a centrifugal supply of air–oil aerosol with simultaneous application of CCA to the grinding zone.

#### 3.2.3. Machined Surface Roughness Analysis

**Figure 6:**
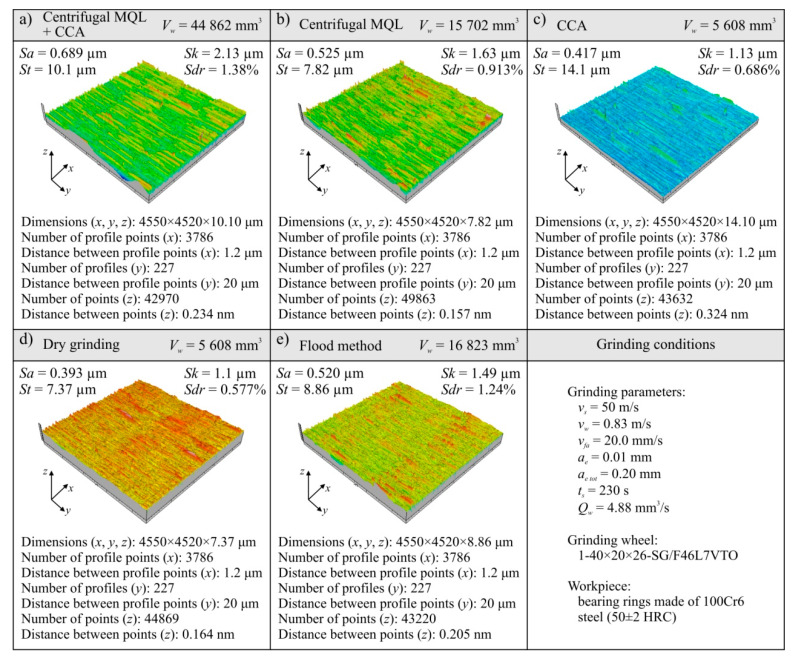
Microtopographs and selected surface roughness parameters of the last ground workpieces from the grinding wheel’s service life: (**a**) hybrid method of cooling and lubrication (centrifugal MQL + CCA); (**b**) centrifugal MQL; (**c**) CCA; (**d**) dry grinding; (**e**) flood method.

**Figure 7:**
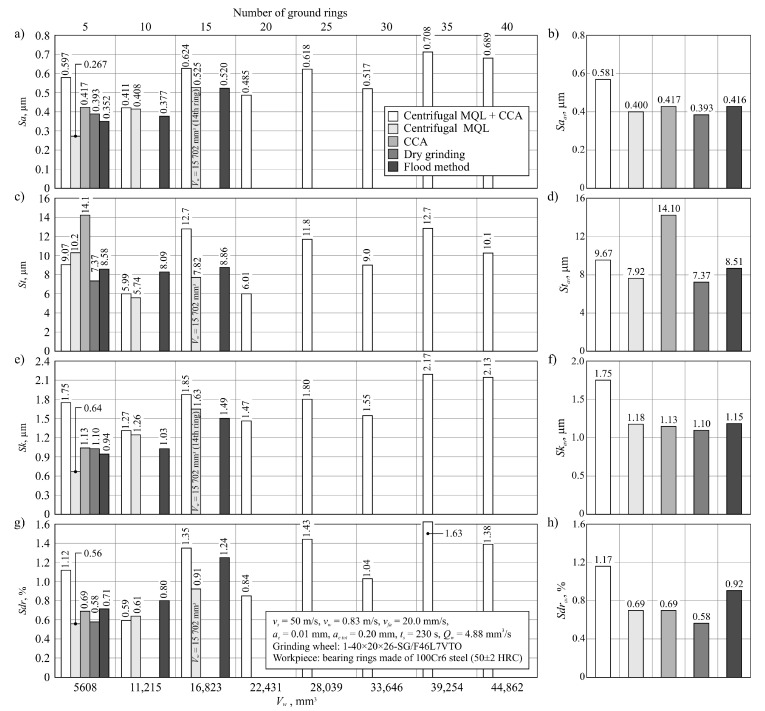
Parameters of the workpiece surface texture (**a**,**c**,**e**,**g**) as well as the average values (**b**,**d**,**f**,**h**) measured after grinding using different conditions of cooling and lubrication: (**a**) arithmetic mean deviation of the surface *Sa*; (**b**) average arithmetic mean deviation of the surface *Sa_av_*; (**c**) total height of the surface *St*; (**d**) average total height of the surface *St_av_*; (**e**) roughness depth of the core *Sk*; (**f**) average roughness depth of the core *Sk_av_*; (**g**) developed interfacial area ratio *Sdr*; (**h**) average developed interfacial area ratio *Sdr_av_*.

The results of the analysis of the machined surface textures for the five analyzed grinding processes are presented in the form of microtopography and a set of values of selected parameters in Figure 6 and Figure 7. Analyses are shown of the last workpiece (ring) from the set of workpieces machined with a given grinding wheel during its durability period, as below:

- For the hybrid method (centrifugal MQL + CCA) this was ring no. 40 (Figure 6a);
- For the centrifugal MQL this was ring no. 14 (Figure 6b);
- For CCA this was ring no. 5 (Figure 6c);
- For dry grinding this was ring no. 5 (Figure 6d);
- For the flood method this was ring no. 15 (Figure 6e).

A summary of the values of selected parameters of machined surface textures is presented in Figure 7, showing changes of arithmetic mean deviation of the surface roughness *Sa*, total height of the surface *St* (Figure 7c), roughness depth of the core *Sk* (Figure 7e), and developed interfacial area ratio *Sdr* (Figure 7g). In addition, Figure 7 presents a comparison of average values of analyzed surface texture parameters for given grinding processes (Figure 7b,d,f,h).

The *Sa* parameter values for all analyzed machined surfaces ranged from 0.267 to 0.708 μm, while the values exceeding the standardized value of 0.63 μm assumed for the surface after grinding were measured only on ring no. 35 and no. 40 for machining under cooling and lubrication conditions using the hybrid method (Figure 7a). This was due to the shaping of the surface by the grinding wheel at the end of its life. This means that for the vast majority of the surfaces ground in the described tests, a surface texture meeting the standard requirements for surfaces after grinding was obtained. This result was obtained with the use of relatively large abrasive grains (no. 46) in the grinding wheels. Such grains were used for the construction of grinding wheels to obtain a porous structure enabling the transport of air–oil aerosol from the inside of the grinding arbor through the intergranular spaces to the grinding zone, which is required with the use of centrifugal MQL. Despite the relatively large grain sizes (average grain size of 46 according to the (Federation of European Producers of Abrasives) FEPA standard equals 0.355–0.425 mm), the SG™ sintered microcrystalline corundum grains used, due to their microstructure, are characterized by being able to shape the workpiece surface with relatively low roughness [64].

Comparing the average values of surface texture parameters for particular machining conditions (Figure 7), it can be seen that slightly higher values were determined on surfaces machined under hybrid method conditions. For the *Sa_av_* parameter, the difference is about 30% in relation to the other methods of cooling and lubrication of the grinding zone (Figure 7b). Such a result should be explained by a much longer grinding wheel working life with the hybrid method. Along with an increase in the volume of removed machined material, the grinding wheel wear also increased, mainly in the form of dulling the vertices of active abrasive grains and bridges of the bond, as well as progressive clogging of these smoothed surfaces with microchips. Systematic increases of the share on the GWAS worn surfaces of active vertices and clogged surfaces increases the intensity of furrows and the formation of outflows (front and side) accompanying the separation of the workpiece material in the form of chips. These phenomena negatively influence the roughness of the machined surface.

Changes in surface roughness under flood conditions should be considered as typical for grinding wheels working with microcrystalline sintered corundum (Figure 7). In the initial period of work on the GWAS there were a lot of sharp microcrystalline cutting microvertices, allowing a surface with relatively low roughness to be to obtained (*Sa* = 0.352 μm for *V_w_* = 5608 mm^3^). The abrasive wear of active vertices in grains that progressed during operation gradually led to a reduction in the number of sharp edges, resulting in an increase in the cross-section of the cutting layer per single cutting vertex. The result was a surface with a higher roughness (*Sa* = 0.520 μm for *V_w_* = 16,823 mm^3^).

During grinding using the centrifugal MQL method, a very similar course of changes to the results obtained in flood method grinding conditions of the workpiece surface texture parameters during grinding wheel operation was recorded (Figure 7). Although the roughness of the machined surface changed similarly, it was shaped in a slightly different way. In the case of minimized delivery of air–oil aerosol (centrifugal MQL), the lubrication function was very effective in reducing the abrasive wear of the abrasive grains. At the same time, the phenomenon of GWAS clogging with grinding products was intense, which can be explained by the uneven oil penetration into the GWAS areas in contact with the workpiece surface.

In the case of dry grinding, the degradation of the GWAS occurred the fastest and the machined surface texture (recorded after machining *V_w_* = 5608 mm^3^ of the workpiece material) was shaped by significantly dull abrasive grains. In this case, the phenomenon of furrowing and the formation of front and side outflows caused by an increased cross-section of the cutting layer per single cutting vertex was accompanied by intensive surface smoothing caused by friction between the GWAS and workpiece. As a result of these phenomena, a surface with a roughness of *Sa* = 0.393 μm was formed (Figure 7).

The way the ground surface was formed in the grinding process carried out under CCA delivery conditions can be interpreted in a very similar way to dry grinding. However, it seems that the process of smoothing out the abrasive grains in this case was somewhat slower due to the CCA cooling effect. As a result, after removal of 5608 mm^3^ of the machined material, the surface roughness described by the *Sa* parameter was recorded at *Sa* = 0.417 μm (Figure 7). However, it turned out that the cooling effect was too small to allow the grinding wheel to work for longer under such conditions.

**Figure 8:**
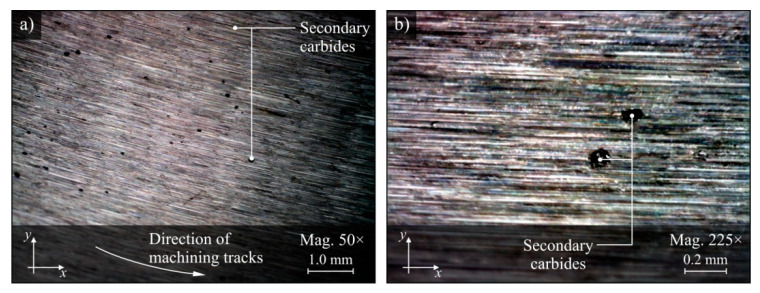
Microscope images of the workpiece surface machined by delivery of cooled compressed air, with visible surface inclusions recorded by the Dino-Lite Edge AM6715MZT digital measuring microscope: (**a**) magnification 50×; (**b**) magnification 225×.

On the surface machined under the conditions of delivery of only CCA to the grinding zone, unusual defects were observed in the form of the point inclusions shown in Figure 8. After hardening, 100Cr6 bearing steel may contain from 5% to 10% of the volume of undissolved secondary carbides in its structure, which together with the martensitic structure gives it considerable hardness (up to 64 HRC) [73]. Therefore, it can be assumed that the observed inclusions are secondary carbides formed as a result of high temperature action on the surface layer of the workpiece.

More detailed analyses concerning the condition of the surface layer of the workpieces were carried out on the basis of X-ray diffraction measurements and are described below.

#### 3.2.4. Assessment of Residual Stress State in the Surface Layers of Ground Workpieces

The state of the residual stress in the surface layers after grinding using five tested process conditions was determined by measuring the state of elastic deformations by X-ray diffraction. An Proto iXRD X-ray diffractometer from Proto Manufacturing Inc. was used, equipped with a copper anode lamp emitting a CrK_α_ characteristic radiation beam with a wavelength of *λ* = 0.154 nm and a diameter of 2 mm. The anode voltage and current were 20 kV and 4 mA, respectively. In order to determine the values of residual stresses, the sin^2^*ψ* method was used, whose basic Equation (1) is valid for a flat stress state:

(1) $$\varepsilon_{\varphi ,\psi }=\frac{1}{2}s_{s}\sigma_{\varphi }\sin^{2}\psi +s_{1}\left(\sigma_{11}+\sigma_{22}\right).$$

The deformation of the crystallographic network *ε_φ,ψ_*, determined by Equation (2), was determined for constant values of the *ψ* angle between −30° and 30°:

(2) $$\varepsilon_{\varphi ,\psi }=\frac{d_{\varphi ,\psi }-d_{0}}{d_{0}},$$

where *d_φ,ψ_* is the interplane distance of fixed network planes measured in the direction defined by angles *φ* and *ψ* in the deformed sample; *d*_0_ is the interplane distance of the same network planes measured in a stress-free sample (*σ* = 0 MPa).

The distance *d*_0_ is replaced by the interplane distance for the angle *ψ* = 0°. From the linear dependence *ε_φ,ψ_* = *f*(sin^2^*ψ*), the value of the straight slope coefficient was determined and the value of stress was calculated according to Equation (3):

(3) $$\sigma_{0}={\left(\frac{E}{1+\nu }\right)}_{hkl}\frac{1}{d_{0}}\left(\frac{\partial d_{\varphi ,\psi }}{\partial \sin^{2}\psi }\right),$$

where *E* is Young’s modulus of elasticity (the calculation assumes the value of *E* = 210 GPa, characterizing steel 100Cr6); *ν* is Poison’s coefficient (assumed value *ν* = 0.30, corresponding to steel 100Cr6).

**Figure 9:**
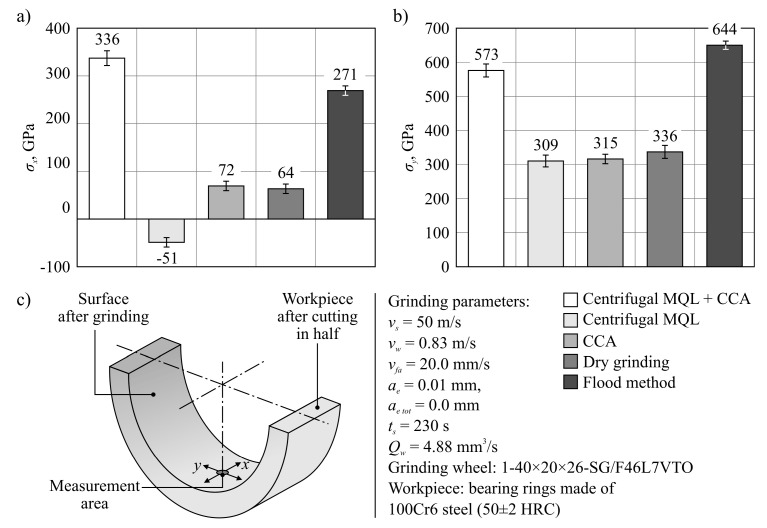
Values of residual stress in the surface layer of workpieces machined with the use of the five tested cooling and lubrication methods of the grinding zone: (**a**) in the *x* direction; (**b**) in the *y* direction; (**c**) diagram illustrating the *x* and *y* measurement directions.

The workpiece rings were cut to allow the head of the Proto iXRD X-ray diffractometer to reach the inner cylindrical surface after grinding. During the measurements, the residual stresses in the surface layer of the workpiece were determined in *x* and *y* directions, with *σ_x_* corresponding to the stresses determined in the direction consistent with the forming cylinder describing the ground ring, while *σ_y_* related to the direction consistent with the base of this cylinder (Figure 9c). Figure 9a,b show the set of determined values of residual stresses *σ_x_* (Figure 9a) and *σ_y_* (Figure 9b) at a depth of about 5 μm in the surface layer of workpieces machined using the five tested methods of cooling and lubrication of the grinding zone.

The last rings from each test cycle, i.e., workpieces processed after different grinding wheel operation times, were selected for the measurement of the residual stresses. In the case of grinding with centrifugal MQL with simultaneous CCA delivery the measurement was made on ring no. 40, for the flood method it was ring no. 15, for centrifugal MQL it was ring no. 10, and ring no. 5 was measured for the other two variants (CCA and dry grinding). Measurements planned in this way do not allow for a unequivocal comparison of the respective variants, which would be reliable in the case of surface layer analysis after an equal machining time. However, the applied strategy of measurements of residual stresses in the surface layer of machined workpieces allowed for a complementary assessment of the quality of the machined surface for the last rings during the life of a given grinding wheel, which was significantly different depending on the conditions of the grinding process.

The analysis of the results of the measurements presented in Figure 9 shows that in the direction of the main grinding movement (rotational movement of the grinding wheel), marked as *y* in the described tests, the residual stresses in the surface layer are positive for all tested workpieces and give values ranging from 309 to 644 MPa (Figure 9b). Tensile stresses recorded at a depth of about 5 μm from the surface are unfavorable after grinding, which is usually the last operation shaping the technological quality of the product, in turn determining its functional quality. Significant values of tensile stress can result in microcracks and exfoliation of the surface during operation, contributing to premature wear of components. In this case, the measured stresses *σ_y_* did not give values that could directly result in defects on the treated surface. A similar order of magnitude of the residual stresses was presented in the description of the grinding results carried out under conditions of limited cooling and lubrication in many works quoted earlier in Section 1 [41,74].

In the *x* direction, which is consistent with the axial feed of the grinding wheel *f_a_*, the values of tensile stresses were significantly lower, while in the case of grinding using the centrifugal MQL method, the measurements result was a negative value (*σ_x_* = −51 GPa), indicating that compressive stresses are considered beneficial from the point of view of exploitation of ground surfaces (Figure 9a). In the remaining variants, *σ_x_* values were positive and ranged from 64 to 336 MPa.

The comparison of measurement results for respective methods of cooling and lubrication of the grinding zone indicates that the most advantageous stress state in the surface layer was obtained in the case of machining with centrifugal MQL (ring no. 10), grinding with CCA delivery (ring no. 5), and dry grinding (ring no. 5). However, it should be remembered that these variants had the shortest wheel life. A much longer tool life is achieved in the case of grinding under flood cooling conditions (15 machined rings) and the hybrid method involving centrifugal MQL with simultaneous CCA delivery (40 machined rings). In this context, the demonstrated differences in the residual stress values should be considered. This is because they mainly result from the quantitative relationship between thermal phenomena and plastic deformation, which depends on the cooling and lubrication conditions of the machining zone, as well as on the GWAS state. A large share of friction generated at the contact points of smoothed vertices of active abrasive grains, ceramic bond bridges, and micro- and macroclogging occurring on grinding wheels with significant wear favors the transfer of more heat to the workpiece. In the case of using the hybrid method combining the centrifugal MQL with CCA delivery, intensive cooling of the area around the direct contact zone of the GWAS with the machined surface by the CAG nozzle also occurs, similar to the flood method conditions. This results in larger temperature drop gradients caused by rapid cooling of the machined surface than is the case with the other three cooling and lubrication varieties included in the tests. As a result, this phenomenon determines the formation of higher values of tensile stresses in the surface layer of the workpieces (Figure 9a,b).

#### 3.2.5. Assessment of the Grinding Wheel Active Surface Condition after Grinding

**Figure 10:**
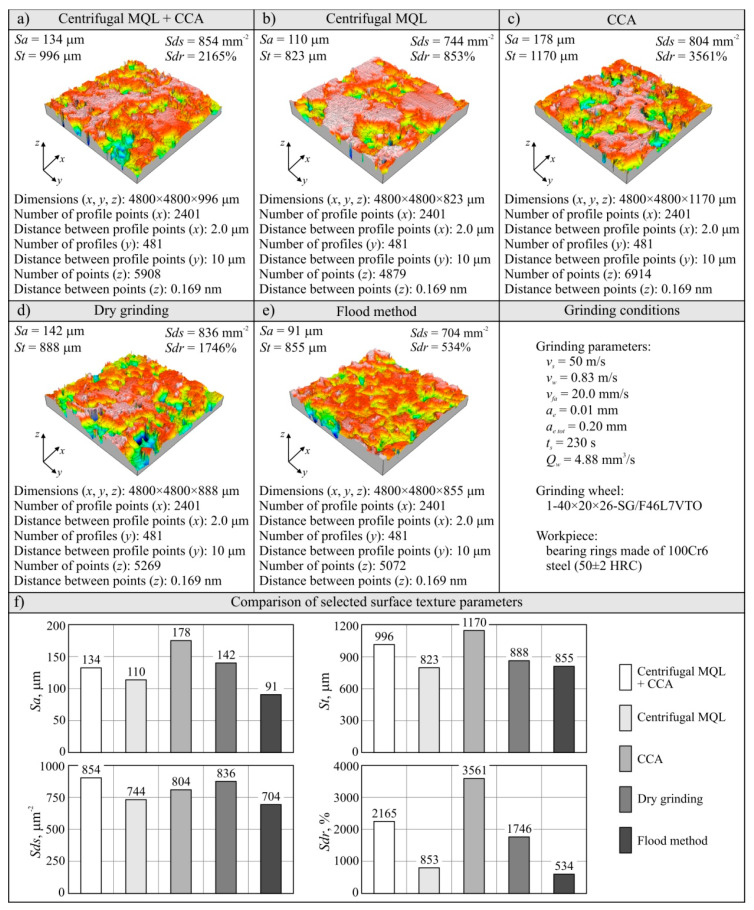
Microtopographs (**a**–**e**) and selected parameters of the geometrical structure of the GWAS (**f**) after grinding with different conditions of cooling and lubrication.

**Figure 11:**
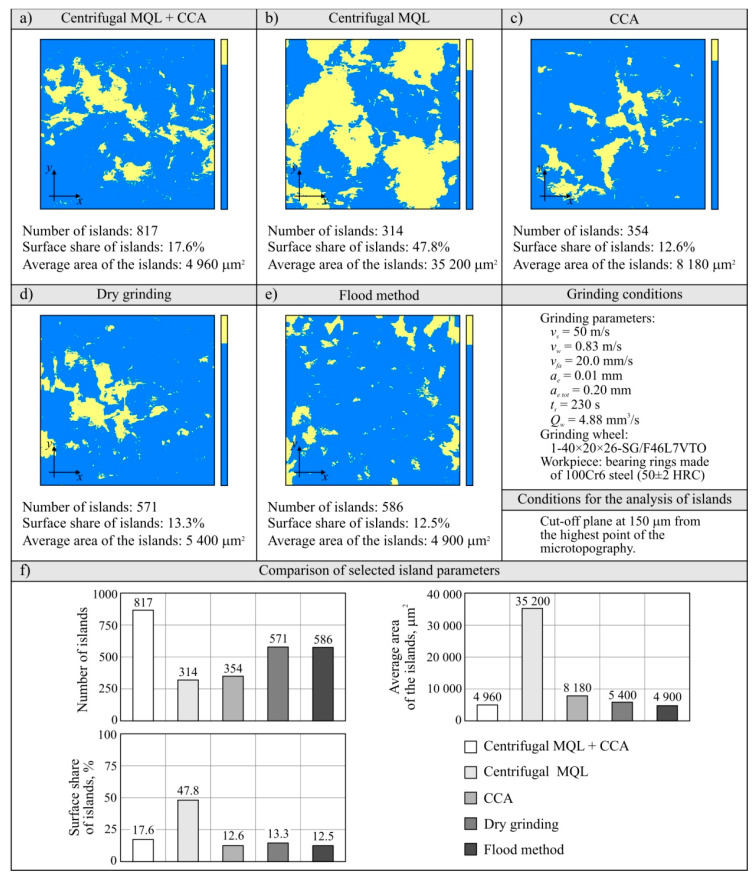
Results of an island analysis (number and area of islands) separated on the GWAS (**a**–**e**), as well as comparison of selected island parameters (**f**).

**Figure 12:**
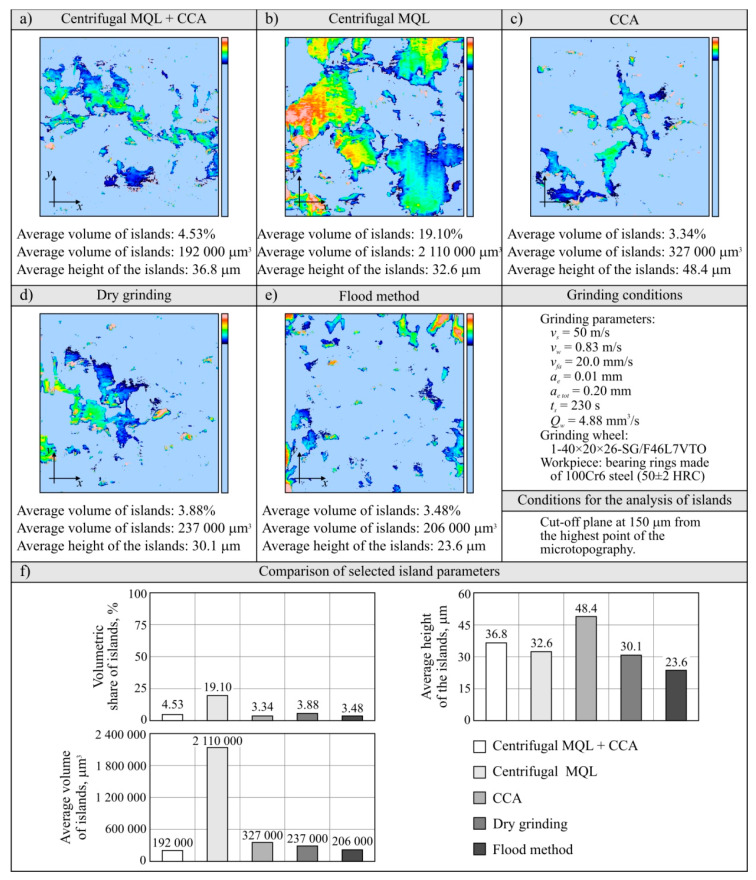
Results of an island analysis (volume and height of islands) separated on the GWAS (**a**–**e**), as well as comparison of selected island parameters (**f**).

**Figure 13:**
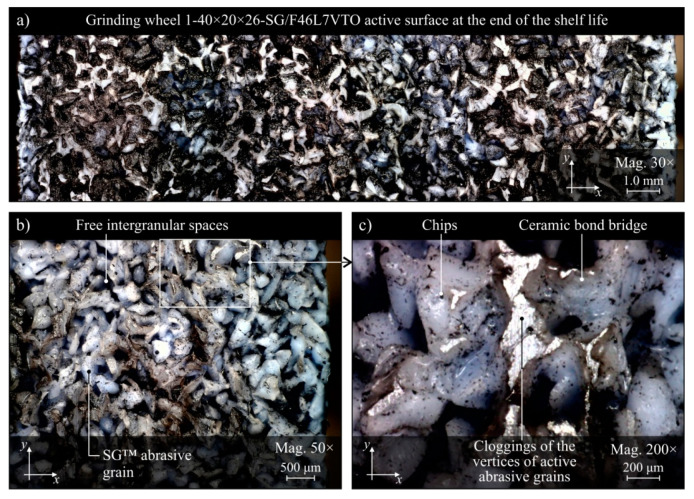
Microscope images of the grinding wheel active surface after machining using the hybrid method of cooling and lubrication (centrifugal MQL + CAG), recorded with the Dino-Lite Edge AM6715MZT digital measuring microscope: (**a**) magnification 30×; (**b**) magnification 50×; (**c**) magnification 200×.

**Figure 14:**
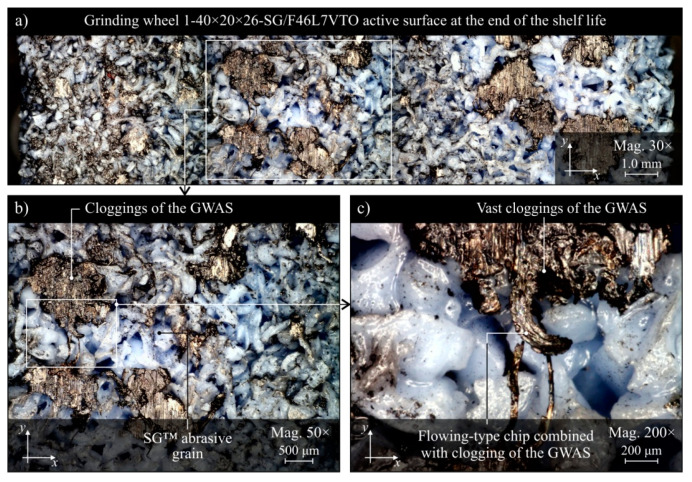
Microscope images of the grinding wheel active surface after machining using centrifugal MQL method of cooling and lubrication, recorded with the Dino-Lite Edge AM6715MZT digital measuring microscope: (**a**) magnification 30×; (**b**) magnification 50×; (**c**) magnification 200×.

**Figure 15:**
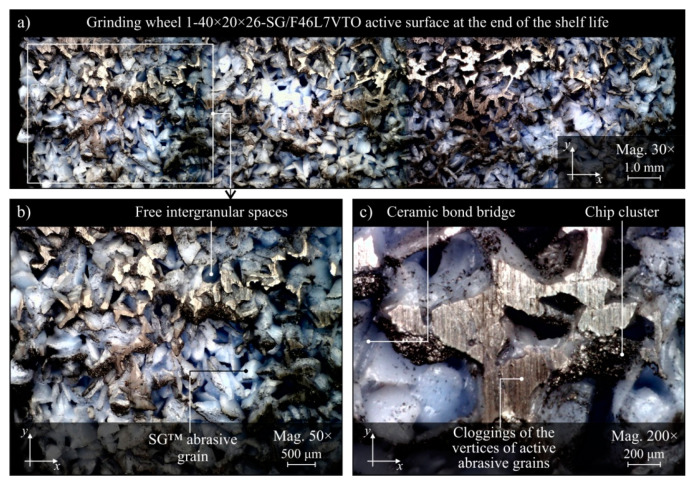
Microscope images of the grinding wheel active surface after machining using CAG for cooling of the machining zone, recorded with the Dino-Lite Edge AM6715MZT digital measuring microscope: (**a**) magnification 30×; (**b**) magnification 50×; (**c**) magnification 200×.

**Figure 16:**
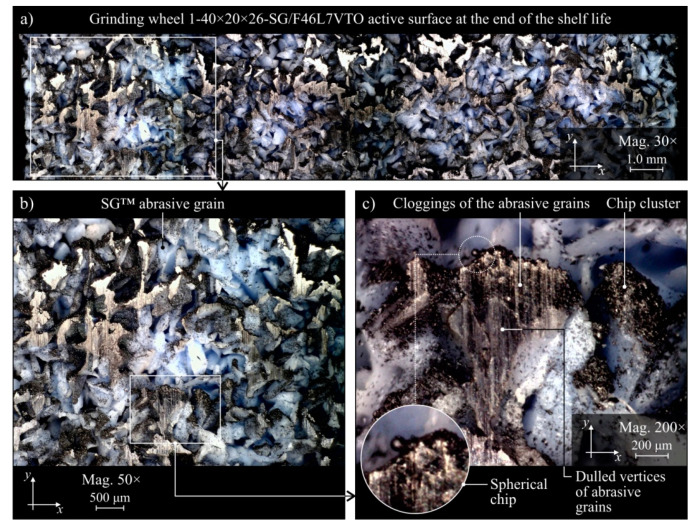
Microscope images of the grinding wheel active surface after machining under dry conditions, recorded with the Dino-Lite Edge AM6715MZT digital measuring microscope: (**a**) magnification 30×; (**b**) magnification 50×; (**c**) magnification 200×.

**Figure 17:**
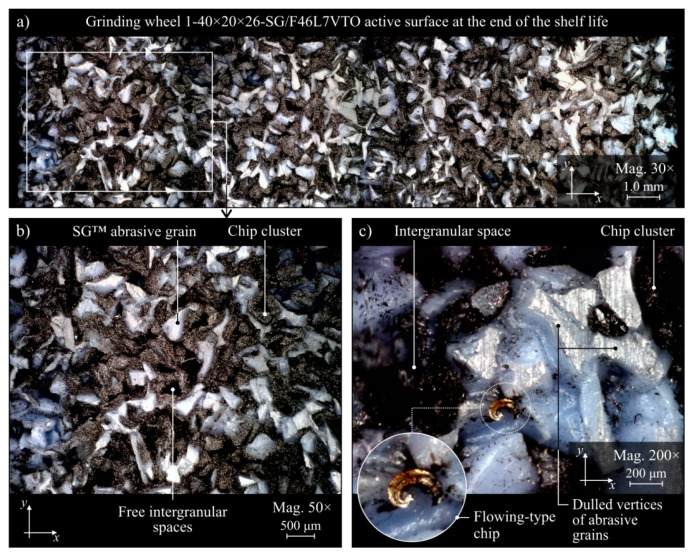
Microscope images of the GWAS after machining using the flood method of cooling and lubrication, recorded with the Dino-Lite Edge AM6715MZT digital measuring microscope: (**a**) magnification 30×; (**b**) magnification 50×; (**c**) m magnification 200×.

The evaluation of the GWAS condition after grinding in the five grinding zone cooling and lubrication methods was carried out on the basis of surface texture measurements with the use of the Talysurf CLI 2000 measuring system. The obtained analysis results are presented in the form of microtopographs in Figure 10a–e. There are also diagrams allowing comparison of the values of selected parameters of the GWAS texture after the end of the service life for the five different grinding processes (Figure 10f). Due to the presence on the evaluated smoothed abrasive grain vertices and clogging areas in the GWAS, an additional analysis of the islands was carried out. The results of the island analysis are presented in Figure 11 with reference to the number and area of islands, while the parameters concerning the volume and height of islands are given in Figure 12. The analysis of islands made it possible to separate the elements in the GWAS, which were above the cut-off plane, defined in this case at the level of 150 μm from the highest point of microtopography. The set of state analyses of the five evaluated GWAS at the end of the service life is complemented by microscopic images recorded with the Dino-Lite Edge AM7915MZT digital microscope (magnification from 30× to 200×), shown in Figure 13, Figure 14, Figure 15, Figure 16 and Figure 17.

The presented microtopographs, as well as the comparison of the values of parameters *Sa*, *St*, *Sds*, and *Sdr*, indicate a different state of texture for the evaluated GWAS. It is noteworthy that after grinding under centrifugal MQL conditions, extensive clogging of grain vertices and intergranular spaces is clearly visible in the GWAS (Figure 10b, Figure 11b, Figure 12b and Figure 14). The clogging resulted in relatively small values of amplitude parameters of roughness (*Sa* and *St*), as well as a reduction in the density of summits of the surface *Sds* and a small value for the developed interfacial area ratio *Sdr* (Figure 10f). Similar values for the evaluated surface texture parameters were obtained on the basis of GWAS measurement after grinding under flood cooling conditions. In this case, extensive clogging was not observed on the GWAS (Figure 10e, Figure 11e and Figure 12e), whereas smoothed vertices of active abrasive grains and numerous chips deposited in intergranular spaces are clearly visible (Figure 17). As a result, this contributed to a reduction in both the total height of the surface roughness *St*, as well as the mean deviation of the surface roughness *Sa*, which caused relatively small values of the *Sds* and *Sdr* parameters (Figure 10f).

The largest share of surface (47.8%) and volume (19.1%) of the islands (corresponding to the areas clogged with chips and other grinding products, as well as abrasive grains) among the evaluated GWAS was characterized by the surface of the grinding wheel working under centrifugal MQL conditions (Figure 11 and Figure 12). There were few instances of clogging, however these were extensive (Figure 14), which translated into by far the highest values for the average surface (Figure 11f) and the average volume of islands (Figure 12f) among the analyzed wheels. For the remaining four wheels, the values of parameters describing islands on the GWAS did not differ significantly. The exception, however, was for the number of islands, which was by far the highest after grinding with the hybrid method (Figure 11f). A large number of islands with a moderate surface (17.6%) and volume (4.53%) resulted in a relatively small average surface area of 4960 μm^2^ and the smallest average island volume of 192,000 μm^3^ (Figure 12f). Such values for the islands parameters indicate the occurrence of numerous but small flattened areas on the evaluated GWAS, which may correspond to blunt cutting vertices and microclogging in the absence of extensive clogging of intergranular spaces (most unfavorable from the point of view of wheel life and machined surface quality). These occurrences on the evaluated surface are confirmed by the results of microscopic observations presented in Figure 13.

It can be assumed that the significant difference between the grinding wheel active surface condition during machining with delivery of air–oil aerosol and CCA (centrifugal MQL + CCA) and a grinding wheel carrying out the machining under similar conditions but without a CAG nozzle results from the chips not being blown out of the grinding zone by the CCA jet and the higher temperature in the latter case. As a result, in the absence of a CCA jet, chips accumulate in the grinding zone and can re-enter between the GWAS and the workpiece surface at different times and in different amounts, contributing to intensive clogging of free intergranular spaces and creating so-called heat spots on the surface of the grinding wheel. This is a serious disturbing factor, limiting the life of the wheel and negatively affecting the quality of the machined surface (thermal defects), the stability of the machining process, and the repeatability of the results obtained in it. The lack of influence of the CCA flux directed both before and directly behind the grinding zone results in a relatively higher machining temperature, which is an additional factor facilitating chip adhesion to GWAS.

Comparison of microscopic images of GWAS after grinding in the five described variants of cooling and lubrication of the grinding zone (Figure 13, Figure 14, Figure 15, Figure 16 and Figure 17) reveals a large amount of oil present on the surfaces of grinding wheels after machining with the centrifugal air–oil aerosol supply system using the MQL method (Figure 13 and Figure 14). This confirms the effective penetration of the lubricant through the open structure of the grinding wheels used. The relatively largest share of dulled cutting vertices of active abrasive grains is visible on the surface of grinding wheels working with CCA flux (Figure 15) and in dry conditions (Figure 16), i.e., without lubricant. Such circumstances are conducive to generating a greater heat flux resulting from friction in the GWAS contact zone with the workpiece surface. This leads to a relatively higher share of plastic flow of abrasive grains under the influence of high temperature and pressure, which is manifested by the smooth surface of the active grains vertices.

Both parametric quantitative evaluation (Figure 10, Figure 11 and Figure 12) and visual qualitative evaluation (Figure 13, Figure 14, Figure 15, Figure 16 and Figure 17) of the five GWASs analysis indicate that grinding wheels working with the hybrid and flood method are in the best condition at the end of their life. It should be stressed that the first of the mentioned grinding wheels (hybrid method) worked over 2.6 times longer, while at the same time the oil expenditure was only 350 mL/h, which is only about 1.46‰ of the oil-in-water emulsion expended in the flood method (*Q_FM_* = 4.0 L/min).

## 4. Conclusions

The wide range of experimental studies presented in this article has shown that it is possible to integrate centrifugal minimum quantity lubrication with the MQL method and cooling with the CCA stream generated by the CAG nozzle in the internal cylindrical grinding process. It has been shown that such a hybrid method effectively supports the cooling function when using air–oil aerosol lubrication and is characterized by the most advantageous grinding results among the five compared solutions in this area. Analyses carried out in relation to a number of criteria for the assessment of the grinding process have led to the following detailed conclusions.

1. The application of the hybrid method allows the longest grinding wheel life among the five compared grinding types, which is about 2.7 times longer than the life of the flood cooled and centrifugal MQL grinding wheel, and as much as 8 times longer than the life of grinding wheels under the conditions of only CCA and dry grinding.
2. In the case of hybrid grinding, the highest grinding ratio value *G* = 150.2 mm^3^/mm^3^ was obtained, which can be explained by the precise centrifugal supply of the lubricant (air–oil aerosol) through the pores of the grinding wheel to the grinding zone, and by the coolant (CCA stream) being directed before and directly behind the grinding zone.
3. The application of simultaneous delivery of the air–oil aerosol and the CCA stream reduced the friction from the dulled grinding wheel vertices, resulting from the effective penetration of oil into the contact zone with the machined surface, while ensuring stable thermal conditions in the grinding zone. This limited the abrasive wear of the vertices of active abrasive grains, preserving their cutting ability over a longer period of operation.
4. Temperature measurements carried out using the thermovision method showed that among the group of NDG methods (centrifugal MQL + CCA, centrifugal MQL, CCA), the newly developed hybrid method can be considered the most advantageous one, for which an approximately 30% lower value of *Θ_w av_* = 70.7 °C and approximately 50% less lower value of *Θ_GWAS av_* = 27.5 °C were obtained in relation to the dry grinding results.
5. Comparison of the texture parameters for particular grinding conditions showed slightly higher values for surfaces machined under cooling and lubrication conditions using the hybrid method, resulting from the significantly longer wheel life, which together with progressive wear, intensified the phenomenon of furrowing and formation of outflows accompanying the separation of the workpiece material in the form of chips, which had a negative effect on the roughness of the ground surface.
6. The measurements of residual stresses on the surface layers of workpieces showed that in the *y* direction, consistent with the rotational movement of the wheel, the residual stresses in the surface layer were positive and gave values ranging from *σ_y_* = 309,644 MPa. In the *x* direction (according to the axial feed of the grinding wheel *f_a_*) the values were significantly smaller, while for the centrifugal MQL method the measurement results were negative value (*σ_x_* = −51 GPa), indicating the presence of compressive stress.
7. The residual stress values in the surface layers of workpieces resulted from differences in cooling and lubrication conditions of the grinding zone; in the case of the hybrid cooling and lubrication method (centrifugal MQL + CCA), there was a phenomenon of intensive cooling of the area around the GWAS contact zone with the workpiece surface (similar to the flood method conditions), causing larger temperature drops resulting from rapid cooling of the machined surface than with the other methods included in the comparison.
8. The quantitative and qualitative evaluation of the grinding of the GWAS with the hybrid method (centrifugal MQL + CCA) has confirmed the positive effect of the CCA flux in preventing the accumulation of chips in the grinding zone and their re-entry between the GWAS and the workpiece surface.

The obtained experimental results are also a partial verification of the results of preliminary numerical simulations conducted by the authors using the computational fluid dynamics method and described in detail in the paper [65]. The results of the CFD simulation showed that with a specific angular setting of two outlets of compressed cold air supply, it is possible to achieve the simultaneous effect of cooling the contact of the GWAS with the machined surface and cleaning chips out of the grinding zone. In the experimental research, the most favorable parameters of the centrifugal MQL-CCA method among the variants analyzed in numerical studies were adopted. The obtained results of the experimental tests confirmed the effectiveness of the implementation of both the lubricating and cooling function, as well as the beneficial effect on the cleaning of the grinding zone from the grinding products, confirming the validity of the assumptions made in the simulation tests.

## Figures and Tables

**Table 1:** Heat capacity values for water, oil, and air [1].

Cooling and Lubrication Method	Type of Medium	Heat Capacity *C_p_*, kJ/kg·K
Flood method (usually 3–5% oil-in-water emulsion)	Water	4.18
MQL (air–oil aerosol)	Oil	1.92
	Air	1.04

**Table 2:** Grinding conditions.

**Process**	Reciprocal, peripheral internal cylindrical grinding				
**Grinding Machine**	Universal grinding machine RUP 28P manufactured by Mechanical Works Tarnów SA				
**Grinding Wheel**	1-40 × 20 × 26-SG/F46L7VTO				
**Grinding Parameters**	*v_s_* = 50 m/s, *v_w_* = 0.83 m/s, *v_fa_* = 20.0 mm/s, *a_e_* = 0.01 mm, *a_e tot_* = 0.20 mm, *t_s_* = 230 s; *Q_w_* = 4.88 mm^3^/s				
**Workpiece**	Internal surfaces of bearing rings made of 100Cr6 steel (50 ± 2 HRC), internal diameter: *d_w_* = 51 mm, width: *b_w_* = 35 mm				
**Cooling and Lubricating**	Centrifugal MQL + CCA	Centrifugal MQL	CCA	Flood method	Dry grinding
**Lubricating Agent**	Centrifugally delivered air–oil aerosol (through a grinding wheel)	Centrifugally delivered air–oil aerosol (through a grinding wheel)	–	5% aqueous solution of Castrol Syntilo RHS oil delivered by the flood method with working pressure of 0.12 MPa and flow rate *Q_FM_* = 4.0 L/min	–
	Head: ZR-K 360°	Head: ZR-K 360°			
	Supply air pressure: 0.6 MPa	Supply air pressure: 0.6 MPa			
	Oil: Cimtech^®^ MQL	Oil: Cimtech^®^ MQL			
	*Q_MQL_* = 350 mL/h	*Q_MQL_* = 350 mL/h			
**Cooling Agent**	Compressed cooled air	–	Compressed cooled air		
	Nozzle: Vortec 610		Nozzle: Vortec 610		
	Supply pressure: 0.6 MPa		Supply pressure: 0.6 MPa		
	*Q_CCA_* = 49.8 dm^3^/min		*Q_CCA_* = 49.8 dm^3^/min		
	Temperature of CCA: −5 °C		Temperature of CCA: −5 °C		

## Nomenclature

*Symbols*	
*a_d_*	dressing allowance, mm
*a_e_*	working engagement (machining allowance), mm
*a_e tot_*	total working engagement (machining allowance), mm
*b_w_*	workpiece width, mm
*C_p_*	heat capacity, kJ/kg·K
*d_0_*	the interplane distance of the same network planes measured in a stress-free sample
*d_s_*	grinding wheel outer diameter, mm
*d_w_*	workpiece internal diameter, mm
*d_φ,ψ_*	the interplane distance of fixed network planes measured in the direction defined by angles *φ* and *ψ* in the deformed sample
*E*	Young’s modulus, GPa
*F*	grinding force, N
*F’*	specific grinding force, N/mm
*fa*	grinding wheel axial feed, mm
*G*	grinding ratio, mm^3^/mm^3^
*i_d_*	number of dressing passes
*n_d_*	grinding wheel rotational speed while dressing, rpm
*P*	grinding power, W
*P_av_*	average grinding power, W
*Q_CCA_*	compressed cold air flow rate, dm^3^/min
*Q_d_*	diamond dresser mass, kt
*Q_FM_*	flood method flow rate, L/min
*Q_MQL_*	MQL method flow rate, mL/h
*Q_w_*	material removal rate, mm^3^/s
*Q’_w_*	specific material removal rate, mm^3^/s·mm
*Ra*	arithmetic mean deviation of the workpiece roughness profile, μm
*Rz*	maximum height of the roughness profile within a sampling length, μm
*Sa*	arithmetic mean deviation of the surface roughness, μm
*Sa_av_*	average arithmetic mean deviation of the surface roughness, μm
*Sdr*	developed interfacial area ratio, %
*Sdr_av_*	average developed interfacial area ratio, %
*Sk*	roughness depth of the core, μm
*Sk_av_*	average roughness depth of the core, μm
*St*	total height of the surface, μm
*St_av_*	average total height of the surface, μm
*t_s_*	grinding time, s
*V_s_*	grinding wheel volumetric wear, mm^3^
*V_w_*	material removal, mm^3^
*v_fa_*	axial table feed speed while grinding, mm/s
*v_fd_*	axial table feed speed while dressing, mm/s
*v_s_*	grinding wheel peripheral speed, m/s
*v_w_*	workpiece peripheral speed, m/s
*v_s_*	grinding wheel peripheral speed, mm/s
*v_w_*	workpiece peripheral speed, mm/s
*Greek symbols*	
*ΔP*	grinding power gain, W
*ΔP_av_*	average grinding power gain, W
*ε*	emission coefficients for infrared thermal imaging measurements
*ε_φ,ψ_*	deformation of the crystallographic network
*λ*	wavelength of the X-ray diffraction method radiation beam, nm
*Θ_GWAS_*	grinding wheel temperature, °C
*Θ_GWAS av_*	average grinding wheel temperature, °C
*Θ_w_*	workpiece temperature, °C
*Θ_w av_*	average workpiece temperature, °C
*φ* and *ψ*	the X-ray diffraction method direction angles,
*σ*	standard deviation
*σ_x_*	residual stresses in the surface layer (*x* direction), MPa
*σ_y_*	residual stresses in the surface layer (*y* direction), MPa
*ν*	Poison’s coefficient
*Acronyms*	
CAG	Cold Air Gun
CAMQL	Cold Air Minimum Quantity Lubrication
CAOM	Cold Air and Oil Mist
CCA	Compressed Cooled Air
CFD	Computational Fluid Dynamics
GWAS	Grinding Wheel Active Surface
MQC	Minimum Quantity Cooling
MQCL	Minimum Quantity Cooling Lubrication
MQL	Minimum Quantity Lubrication
